# Complexation design of cationized gelatin and molecular beacon to visualize intracellular mRNA

**DOI:** 10.1371/journal.pone.0245899

**Published:** 2021-01-25

**Authors:** Sho Takehana, Yuki Murata, Jun-ichiro Jo, Yasuhiko Tabata

**Affiliations:** Laboratory of Biomaterials, Institute for Frontier Life and Medical Sciences, Kyoto University, Kyoto, Japan; Cardiff University, UNITED KINGDOM

## Abstract

The objective of this study is to prepare cationized gelatin-molecular beacon (MB) complexes for the visualization of intracellular messenger RNA (mRNA). The complexes were prepared from cationized gelatins with different extents of cationization and different mixing ratios of MB to cationized gelatin. The apparent size of complexes was almost similar, while the zeta potential was different among the complexes. Irrespective of the preparation conditions, the complexes had a sequence specificity against the target oligonucleotides in hybridization. The cytotoxicity and the amount of complexes internalized into cells increased with an increase in the cationization extent and the concentration of cationized gelatin. After the incubation with complexes prepared from cationized gelatin with the highest extent of cationization and at mixing ratios of 10 and 20 pmole MB/μg cationized gelatin, a high fluorescent intensity was detected. On the other hand, the complex prepared with the mixing ratio at 20 pmole/μg did not show any cytotoxicity. The complex was the most effective to visualize the glyceraldehyde-3-phosphate dehydrogenase (GAPDH) mRNA endogenously present. In addition, even for enhanced green fluorescent protein (EGFP) mRNA exogenously transfected, the complex permitted to effectively detect it as well. It is concluded that both the endogenous and exogenous mRNA can be visualized in living cells by use of cationized gelatin-MB complexes designed.

## Introduction

Recently, biological and medical applications of cell potential and functions have been extensively studied. For example, in the field of regenerative medicine, cells with high potentials, such as induced pluripotent stem cells (iPSC) and mesenchymal stem cells (MSC), have been paid attention. With human matured and functional cells differentiated from iPSC, various disease-specific models have been designed and prepared *in vitro*. The models enable the researches of drug safety and activity evaluation [1–4]. It has been demonstrated that MSC have high anti-inflammatory, anti-fibrotic and immunoregulation abilities. Based on these properties, the transplantation therapy are being conducted to show a high therapeutic efficacy in diseases [5–7]. Under these circumstances, it is of prime importance to develop the imaging technologies and methodologies to visualize the biological functions of living cells for the further development of basic cell biology, drug discovery, cancer diagnosis, and regenerative medicine.

Generally, the biological function and state of cells are regulated by the level or the time point of messenger RNA (mRNA) expression and the consequent proteins synthesis. Thus, it may be powerful for the evaluation of cell functions to detect the mRNA level. However, by the polymerase chain reaction (PCR) conventionally used, it is technically difficult to detect mRNA in living cells because the destruction of cells is required for the assay. In addition, since the assay result reflects a population of cells, it is theoretically impossible to detect the mRNA expression change of each cell. It is also difficult to evaluate the mRNA expression of a specific cell type in the co-culture system. In this circumstance, a technique to detect mRNA in real time without cell destruction is expected to explore.

Molecular beacon (MB) is an imaging probe to detect the nucleic acids and has been extensively used in the research fields of nucleic acid chemistry [8–14]. MB is a nucleic acid derivative with a stem loop structure, and a quencher and a fluorophore are conjugated with both the end sides of MB. The loop structure is the complementary sequences of target mRNA. In the absence of the target mRNA, the MB is in the quenched state. On the other hand, in the presence of target mRNA, the structure of MB is changed to be fluorescent because of the hybridization with the target mRNA. It has been reported that several physical techniques used for gene trasnfection, such as electroporation [15, 16] and microinjection [17], are available to the MB delivery into cells. The techniques allow MB to directly internalize into the cytosol, while some specialized instruments are required. There are some cases, where it is difficult to deliver MB to every cells, and the cell viability is low. On the other hand, gold nanoparticles [18–20], dendrimers [21], cationized liposomes [22, 23], and cationized polymers [24–26] have been investigated as the carrier materials of MB. However, in most researches, the MB function is mainly investigated, but the properties of carrier materials-MB complexes, such as the cytotoxicity, the internalization into cells, and the detection efficiency of fluorescence, have not been always investigated well although they are important.

Gelatin is the biocompatible polymer biodegraded by enzymes and has been extensively used for food, drug ingredients, and medical materials. The biosafety of gelatin has been proven through its long-term practical applications. In addition, since gelatin has amino groups or carboxyl groups, it can be chemically modified. Cationized gelatin was prepared by simply introducing of amino compounds to the carboxyl groups of gelatin. Cationized gelatin is readily complexed with nucleic acid molecules, such as plasmid DNA (pDNA) [27, 28], small interfering RNA [29] (siRNA), and MB [25], which can internalize them into cells. In this study, a cationized derivative of gelatin is focused to allow MB to effectively internalize into cells and subsequently detect the target mRNA.

The objective of this study is to evaluate the fluorescent detection property of cationized gelatin-MB complexes prepared in various conditions. Cationized gelatin with varied cationic extents was prepared for the complexation of MB. The complexes are prepared with different mixing ratios of MB to cationized gelatin to assess their physicochemical and hybridization properties. The cytotoxicity, the amount of cell internalization, and the intracellular fluorescence of complexes were investigated. To evaluate the fluorescent detection of MB, both an endogenous mRNA of glyceraldehyde-3-phosphate dehydrogenase (GAPDH) due to the constant expression and the mRNA of enhanced green fluorescent protein (EGFP) exogenously transfected, were used to optimize the preparation condition of the complexes in terms of MB visualization. In this study, KUM6 cells, a line of MSC, were used to evaluate the technological potential of MB-based imaging in MSC which are generally applied to the field of cell transplantation.

## Materials and methods

### Materials

Gelatin with an isoelectric point of 9.0 and the weight-averaged molecular weight of 99,000, prepared by an acidic treatment of pig skin collagen, was kindly supplied from Nitta Gelatin Inc., Osaka, Japan. Molecular beacons (MB) composed of DNA bases for mouse messenger RNA (mRNA) of glyceraldehyde-3-phosphate dehydrogenase (GAPDH) and enhanced green fluorescent protein (EGFP) were synthesized by Integrated DNA Technologies, Inc., Coralville, IA, USA. The sequences of MB [26, 30] were as follows, GAPDH MB: 5’-[TYE^Ⓡ^665]-CTGGTAATCCGTTCACACCGACCTTCACCAG-[Iowa Black^Ⓡ^RQ]-3’; EGFP MB: 5’-[TYE^Ⓡ^665]-ACGCCTTCTCGTTGGGGTCTTTGCTCGGCGT -[Iowa Black^Ⓡ^RQ]-3’ (underline indicates the stem structure). Target oligonucleotides of DNA for GAPDH MB (specific and non-specific sequences) were synthesized by Hokkaido System Science Co., Ltd, Sapporo, Japan. The sequences of specific and non-specific targets were as follows, specific: 5’-TGGTGAAGGTCGGTGTGAACGGATT-3’; non-specific: 5’-TTTCTGAATGGCCCAGGT-3’. Spermine was purchased from Sigma-Aldrich Inc., St Louis, MO, USA. Concentrated hydrochloric acid (HCl) and 1-ethyl-3-(3-dimethylaminopropyl) carbodiimide hydrochloride (EDC) were purchased from Nacalai Tesque. Inc., Kyoto, Japan. The reagents were used without any purification.

### Preparation of cationized gelatin

Cationized gelatin was prepared according to the method previously reported [27, 28]. Briefly, spermine was added at molar ratios of 1, 3, 5, 10, 20, and 50 to the carboxyl groups of gelatin into 50 ml of double-distilled water (DDW) containing 2.0 g of gelatin. The solution pH was adjusted to 5.0 with 11 M HCl. EDC was added at the molar ratio of 3 to the carboxyl groups of gelatin before the addition of DDW into solution to adjust the total volume of 100 ml. Then, the solution was stirred at 40°C for 18 hr and dialyzed against DDW for 3 days at room temperature. The solution was freeze-dried to obtain cationized gelatins. To determine the percentage of spermine introduced into the carboxyl groups of gelatin, the conventional 2,4,6-trinitrobenzene sulfonic acid (TNBS, FUJIFILM Wako Pure Chemical Inc., Osaka, Japan) method was performed [31]. The names of cationized gelatin (percent introduced: 3.17, 16.9, 25.6, 35.4, 42.9, and 55.2 mole%) were defined as SM1, SM3, SM5, SM10, SM20, and SM50, respectively.

### Preparation of cationized gelatin-MB complex

The complexation of cationized gelatin and MB was performed by simply mixing cationized gelatin solution and MB in DDW [30] at different mixing ratios (5, 10, 20, 40, and 200 pmole MB/μg cationized gelatin). The mixed solution was incubated for 15 min at room temperature to obtain the cationized gelatin-MB complexes. The apparent size of complexes resuspended in 10 mM phosphate buffered saline solution (PBS, pH7.4) was measured by dynamic light scattering (DLS, Zetasizer Nano-ZS, Malvern Instruments Ltd., Worcestershire, UK). On the other hand, the zeta potential of complexes resuspended in 10 mM phosphate buffer solution (PB, pH7.4) was measured by electrophoresis light scattering (ELS, Zetasizer Nano-ZS, Malvern Instruments Ltd., Worcestershire, UK). The measurements were independently performed three times unless otherwise mentioned.

### Radiolabeling of MB

MB was labeled with ^125^I, as the methods previously reported [30]. Briefly, MB (50 μM, 5 μl) was incubated at 60°C for 50 min with 2 μl of 0.3 mM Na₂SO₃, 5 μl of Na^125^I, and 5 μl of 4 mM TlCl_3_. A mixture of 100 μl of 0.1 M Na_2_SO_3_, 900 μl of 0.1 M NaCl, 50 mM of Tris, and 1 mM ethylenediaminetetraacetic acid (EDTA) was added to the solution. After the incubation for 30 min at 60°C, the mixture was applied on the PD-10 column (GE Healthcare Bio-Sciences Corp. Piscataway, NJ) to remove the free ^125^I by the gel filtration. The radio activity of ^125^I was measured with a gamma counter (Auto Well Gamma System ARC-380 CL, Aloka Co., Ltd, Tokyo, Japan).

### Hybridization assay

Various concentrations of target oligonucleotides (GAPDH specific or non-specific sequence, 0, 50, 100, 500, 1000, and 5000 nM) and free GAPDH MB or the complexes were mixed in PBS containing 0.5 mM of MgCl_2_ and 0.9 mM CaCl_2_. The mixture was incubated for 1 hr at room temperature protected from light. The fluorescent intensity was measured by Multi-mode Microplate Reader (SpectraMax i3x, Molecular Devices Japan Co., Ltd., Tokyo, Japan). The fluorescent intensity was normalized by that of free MB or complex without the target incubation (0 nM).

### Cell culture experiments

KUM6 cells (JCRB1202) of a mouse bone marrow-derived mesenchymal stem cell line were purchased from JCRB Cell Bank (National Institute of Biomedical Innovation, Health and Nutrition, Osaka, Japan) [32]. The cells were cultured in Iscove’s Modified Dulbecco’s Medium (IMDM, Thermo Fisher Scientific Inc., MA, USA) containing 10 vol% bovine fetal calf serum (FCS, GE healthcare Life Sciences Hyclone laboratories inc., Logan, UT, USA) and 1 vol% penicillin and streptomycin (Nacalai Tesque. Inc., Kyoto, Japan) at 37°C in a 5% CO₂-95% air atmospheric condition. The cells were detached with 0.25 wt% trypsin-containing 1 mM EDTA solution (Nacalai Tesque. Inc., Kyoto, Japan) and continued to culture in 100 mm cell culture dish (Corning Inc., Corning, NY, USA) to allow to grow until to 80% confluency.

### Evaluation of cell viability after incubation with complexes

Cells were seeded to each well of 96 well multi-dish culture plate (Corning Inc., Corning, NY, USA) at a density of 1×10⁴ cells/well and cultured for 24 hr. Then, the medium was changed to the OPTI MEM (Thermo Fisher Scientific Inc., MA, USA), followed by the addition of complexes prepared at different mixing ratios (5, 10, 20, 40, and 200 pmole MB/μg cationized gelatin) to each well. The final concentration of GAPDH MB was 200 nM. After the incubation for 3 hr, 10 μl of 2-(2-methoxy-4-nitrophenyl)-3-(4-nitrophenyl)-5-(2,4-disulfophenyl)-2H-tetrazolium (WST-8) solution was added to each well and further incubated for 1 hr. The absorbance of samples at 450 nm was measured by the plate reader (SpectraMax i3x, Molecular Devices Japan Co., Ltd., Tokyo, Japan). The percentage of cell viability was expressed as 100% for cells without incubation of complexes.

### Evaluation of cell internalization

The cells were similarly seeded on each well of 6 well multi-dish culture plate (Corning Inc., Corning, NY, USA) at a density of 5×10⁴ cells/well and cultured for 24 hr. The medium was changed to OPTI MEM before the complexes prepared at different mixing ratios (5, 10, 20, 40, and 200 pmole MB/μg cationized gelatin) were added. The final concentration of GAPDH MB was 200 nM. After the incubation for 3 hr, the cells were washed with PBS, trypsinized, and collected by centrifugation of 2000 g for 3 min at room temperature. Then, the radioactivity of ^125^I-labeled MB in the cells was evaluated by the gamma counter. On the other hand, the number of cells after the incubation with the complexes was evaluated by the DNA assay. Briefly, the cell lysates prepared by processing with sodium lauryl sulfate (SDS) were mixed with Hoechest solution (Bisbenzimide H33258 Fluorochrome Trihydrochloride DMSO Solution, Nacalai Tesque. Inc., Kyoto, Japan), and then the fluorescent intensity was similarly measured by the plate reader.

### Evaluation of intracellular fluorescence and imaging of endogenous GAPDH mRNA

The cells were seeded on a glass-bottom dish of 35 mm diameter (Matsunami Glass Industries, Ltd., Tokyo, Japan) at a density of 5×10⁴ cells/dish and cultured for 24 hr. The medium was changed to OPTI MEM, and cells were incubated with complexes prepared at different mixing ratios (5, 10, 20, 40, and 200 pmole MB/μg cationized gelatin) for 3 hr. The final concentration of GAPDH MB was 200 nM. After the further incubation of 24 hr, the cells were observed by a fluorescent microscopy BZ-X700 (KEYENCE Co., Ltd., Osaka, Japan) with a 20× objective lens. To quantitatively evaluate the fluorescent intensity of MB, flow cytometry analysis was performed. The cells were washed by PBS to remove the dead cells, and collected by the trypsinization and suspended in PBS. The cell suspension was analyzed on fluorescence activated cell sorting FACSCanto II (Becton Dickinson, Franklin Lakes, NJ, USA) by counting 10,000 cells. On the other hand, to evaluate the intracellular localization of MB, cells which had been incubated with the complex prepared at 20 pmole MB/μg cationized gelatin, were fixed with 4 vol% paraformaldehyde for 20 min, and then the nuclei of cells were stained with 4’,6-diamidino-2-phenylindole (DAPI, 300 nM, Thermo Fisher Scientific Inc., MA, USA) for 10 min. The fluorescent images were taken by the fluorescent microscopy with an oil-immersed 100× objective lens.

### Fluorescent imaging of EGFP mRNA exogenously transfected

The cells were similarly seeded on the glass-bottom dish and cultured for 24 hr. Then, the complex prepared at 20 pmole/μg were added (final 200 nM EGFP MB) at the same procedure described above. As a control, the direct delivery of MB to the cytosol via a membrane fusion was performed by using a transfection reagent: Hemagglutinating Virus of Japan (HVJ)-envelope (HVJ-E) (GenomONE^TM^-Si, ISHIHARA SANGYO KAISHA, LTD, Osaka, Japan) according to the manufacture’s instruction. After the incubation with complexes and HVJ-E for 3 hr, the mRNA of EGFP (5000 ng/dish, TriLink BioTechnologies, Inc., San Diego, CA, USA) was transfected by Lipofectamine^Ⓡ^ MessengerMAX^TM^ (Thermo Fisher Scientific Inc., MA, USA) according to the manufacture’s protocol. Then, the cells were observed by the fluorescent microscopy with the oil-immersed 100× objective lens 36 hr later.

### Statistical analysis

The statistical data were expressed as the mean ± standard deviations (SD). All experiments were performed more than three times to confirm the reproducibility. All the data were analyzed by one analysis of variance (ANOVA) with a post-hoc Tukey-Kramer multiple comparison test on a software StatView version 5.0 (SAS Institute Inc., Cary, NC, USA). *P*-values less than 0.05 were considered to be statistically significant.

## Results

### Preparation of cationized gelatin

In this study, cationized gelatin was prepared as the career material to deliver MB into cell. Table 1 shows the percentage of spermine introduced into the carboxyl groups of gelatin. The percent introduced increased with the increase of spermine addition. In the following experiments, three types of cationized gelatin (SM1, SM10, and SM50) with different extents of cationization were used. The percentages of spermine introduction (cationization extent) were 3.17, 35.4, and 55.2 mole% for SM1, SM10, and SM50, respectively.

**Table 1:** Preparation of cationized gelatin.

Name of cationized gelatin	Molar ratio^a^	Percent introduced
SM1	1	3.17 ± 0.39^b^
SM3	3	16.9 ± 1.55
SM5	5	25.6 ± 1.26
SM10	10	35.4 ± 2.56
SM20	20	42.9 ± 2.54
SM50	50	55.2 ± 3.23

^a^ Molar ratio of spermine added to the carboxyl groups of gelatin in preparation.

^b^ Average ± SD.

### Characterization of complexes

DLS and ELS measurements were performed to characterize the complexes. Tables 2 and 3 show the apparent size and the zeta potential of complexes prepared at different mixing ratios of MB/cationized gelatin, respectively. The apparent size was almost similar, irrespective of the mixing ratio and the cationization extent, while it was around 300 nm. On the other hand, the zeta potential of complexes was influenced by the preparation conditions. The apparent size and zeta potential of complexes prepared at 200 pmole MB/μg cationized gelatin were not detected.

**Table 2:** Apparent size of complexes prepared.

	Apparent size of complexes (nm)		
Mixing ratio (pmole/μg)^a^	SM1	SM10	SM50
5	286.0 ± 39.9^b^	290.3 ± 38.2	302.3 ± 67.0
10	307.3 ± 53.5	306.1 ± 63.4	299.2 ± 75.4
20	280.8 ± 38.7	307.9 ± 39.1	315.8 ± 28.5
40	309.3 ± 7.50	303.2 ± 32.4	325.3 ± 32.4
200	N.D.^c^	N.D.	N.D.

^a^ The amount of MB per 1 μg of cationized gelatin.

^b^ Average ± SD.

^c^ Not detected.

**Table 3:** Zeta potential of complexes prepared.

	Zeta potential of complexes (mV)			
Mixing ratio (pmole/μg)^a^	SM1	SM10	SM50	
5	4.47 ± 0.57 ^b^	8.78 ± 0.27	9.00 ± 0.52	
10	3.59 ± 0.22	9.23 ± 0.12	8.31 ± 0.61	
20	- 5.35 ± 1.53	8.41 ± 0.10	8.58 ± 0.35	
40	- 15.0 ± 1.11	- 14.4 ± 1.38	- 21.0 ± 1.66	
200	N.D.^c^	N.D.	N.D.	

^a^ The amounts of MB per 1 μg of cationized gelatin.

^b^ Average ± SD.

^c^ Not detected.

### Hybridization specificity of complexes

**Fig 1:**
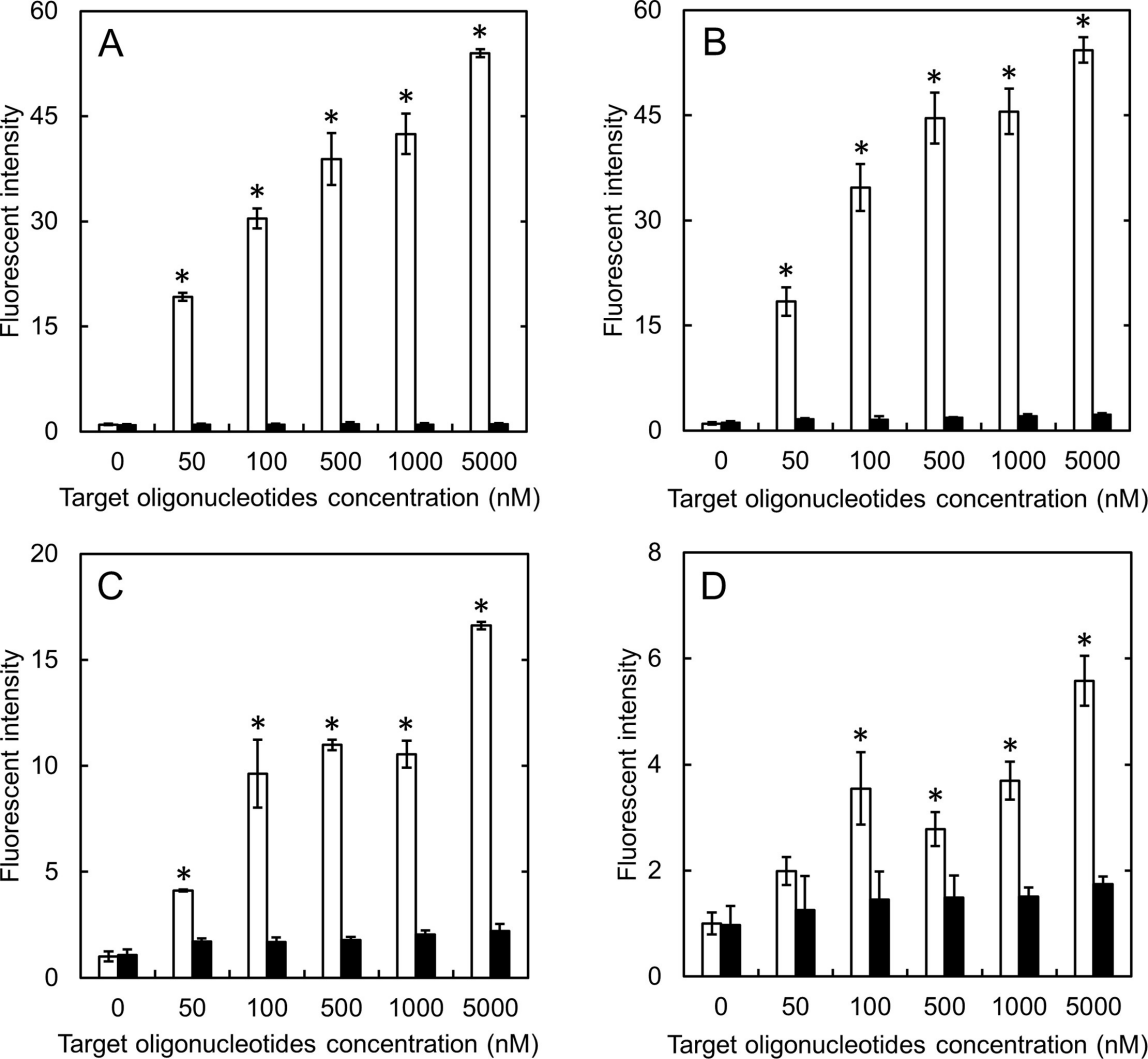
Hybridization specificity of free MB and MB incorporated in complexes. The fluorescent intensity of free MB (A), SM1 (B), SM10 (C), and SM50 complexes (D) mixed with different concentrations of specific (□) and non-specific target oligonucleotides (■). The concentration of GAPDH MB is 100 nM. *, p < 0.05; significant against the fluorescent intensity of non-specific target at the corresponding concentration.

The hybridization specificity of free MB and complex was evaluated (Fig 1). The fluorescent intensity of free MB increased with an increase of the specific target concentration. On the contrary, when incubated with the non-specific target, the fluorescent intensity remained as low as that without the target incubation. For the complexes prepared with different cationized gelatins (Fig 1B–1D), the fluorescent intensity incubated with the specific target became low as the increase of cationization extent. However, the fluorescent intensity incubated with the non-specific target was as low as free MB without target oligonucleotides for any type of complexes.

### Cell viability and MB internalization after incubation with complexes

**Fig 2:**
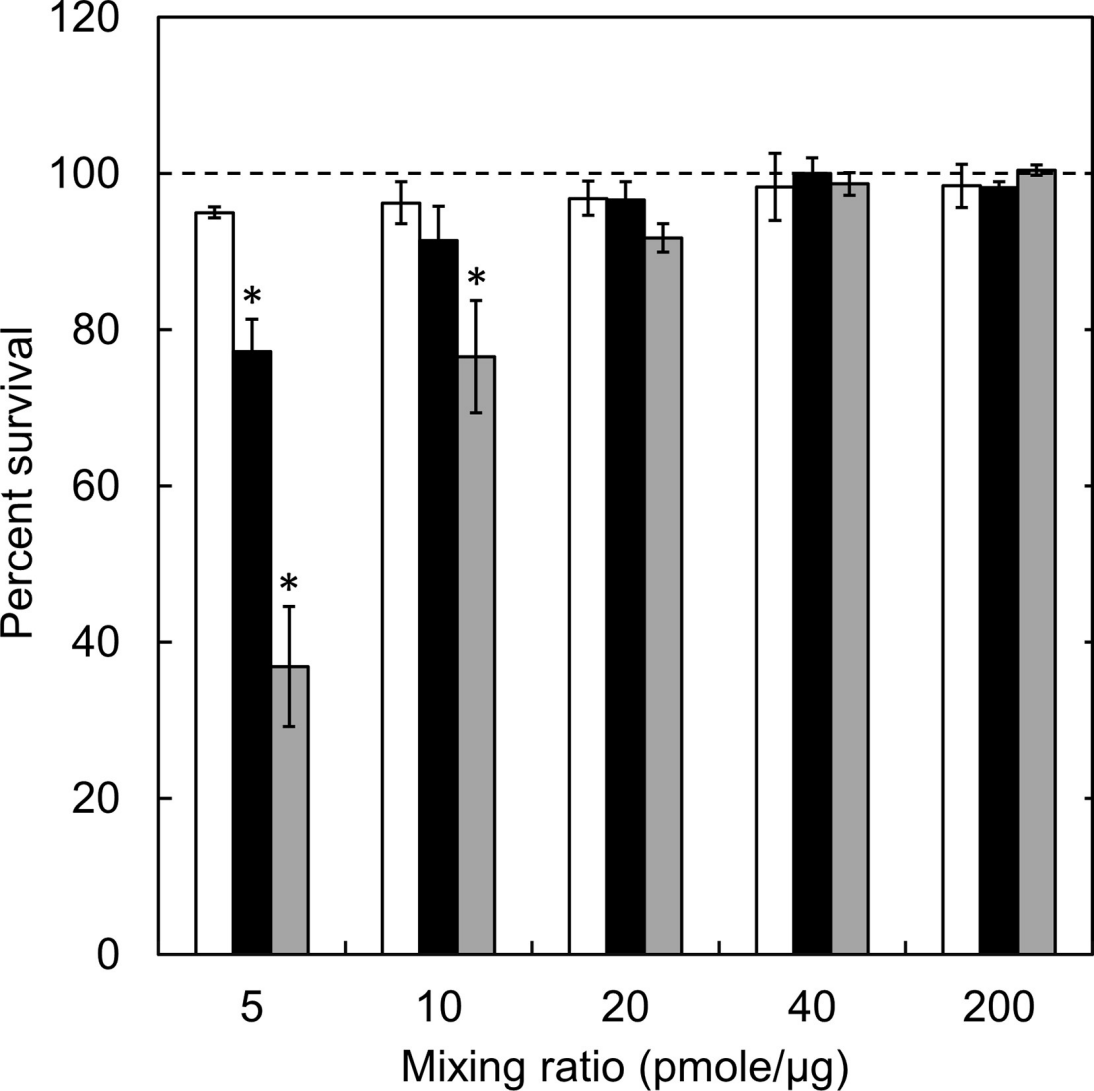
Viability of cells incubated with complexes prepared at different mixing ratios. The cells were incubated with 1, 5, 10, 20, and 40 μg/ml SM1 (□), SM10 (■), and SM50 complexes (■). The GAPDH MB concentration is 200 nM. The viability of cells incubated without complex is expressed as 100%. *, p < 0.05; significant against the percent survival of cells incubated without complexes at the corresponding mixing ratio.

Following the cell incubation with the complexes, the cell viability and MB internalization were evaluated. Fig 2 shows the viability of cells incubated with complexes. The percent survival decreased with a decrease of the mixing ratio, irrespective of the complex type. In addition, the higher cytotoxicity of complexes was observed for the higher extent of cationization. Significant cytotoxicity was observed at 5 and 10 pmole/μg of SM50 complexes and 5 pmole/μg of SM10 complex. For other samples, any cytotoxicity was not seen.

**Fig 3:**
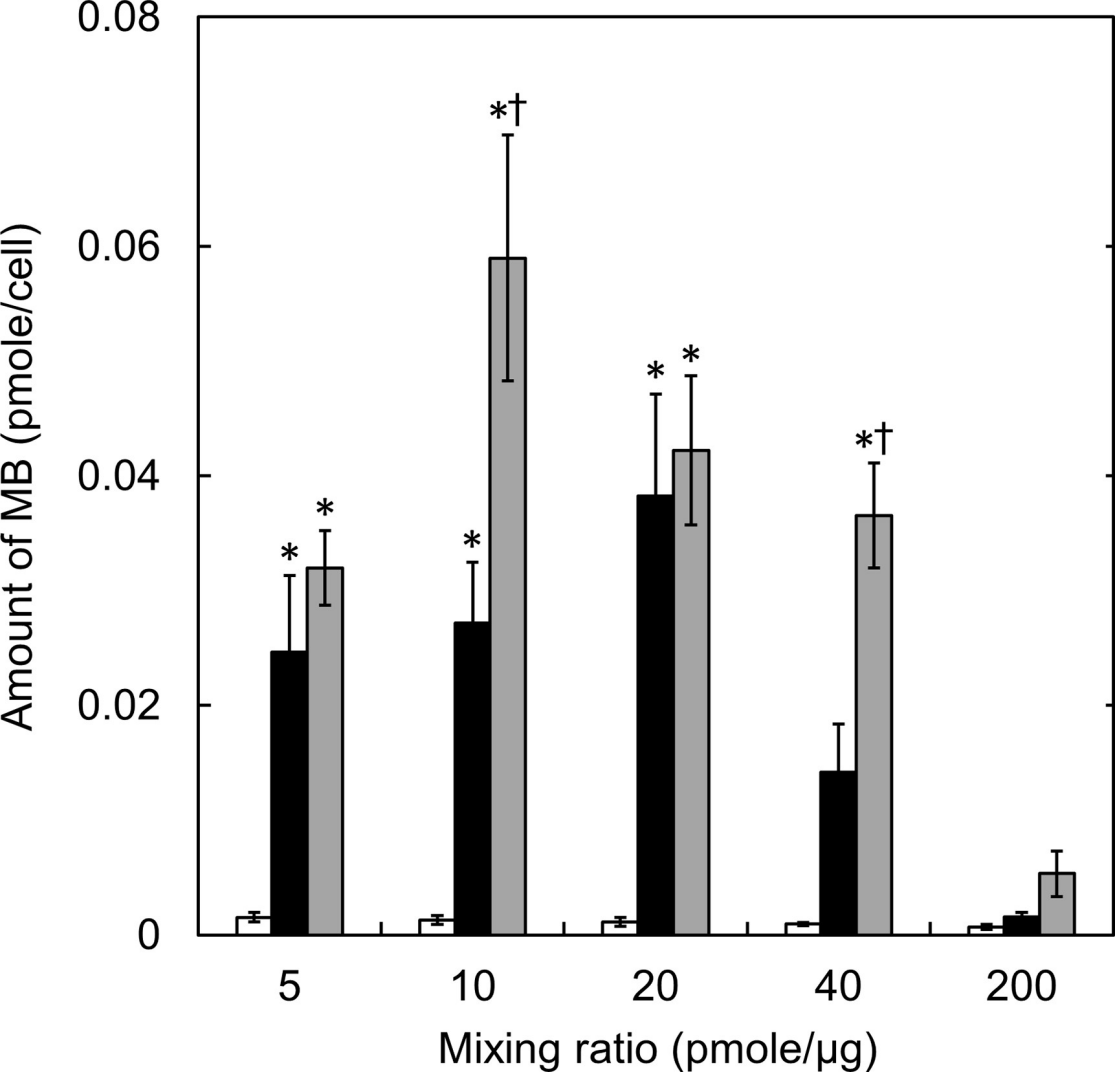
The amounts of MB internalized into cells incubated with complexes prepared at various conditions. The cells were incubated with SM1 (□), SM10 (■), and SM50 complexes (■) prepared at different mixing ratios. The GAPDH MB concentration is 200 nM. *, p < 0.05; significant against the fluorescent intensity of SM1 complexes at the corresponding mixing ratio. †, p < 0.05; significant against the fluorescent intensity of SM10 complexes at the corresponding mixing ratio.

The amounts of complexes internalized into cells were evaluated as a function of mixing ratio (Fig 3). For the SM10 and SM50 complexes, the amounts increased and then decreased as the mixing ratio of complexes increased. The highest amount of MB internalized was observed for the SM50 complex prepared at 10 pmole MB/μg cationized gelatin. On the contrary, for the SM1complex, few complexes were internalized into cells.

### Evaluation of intracellular fluorescence and imaging of endogenous GAPDH mRNA

**Fig 4:**
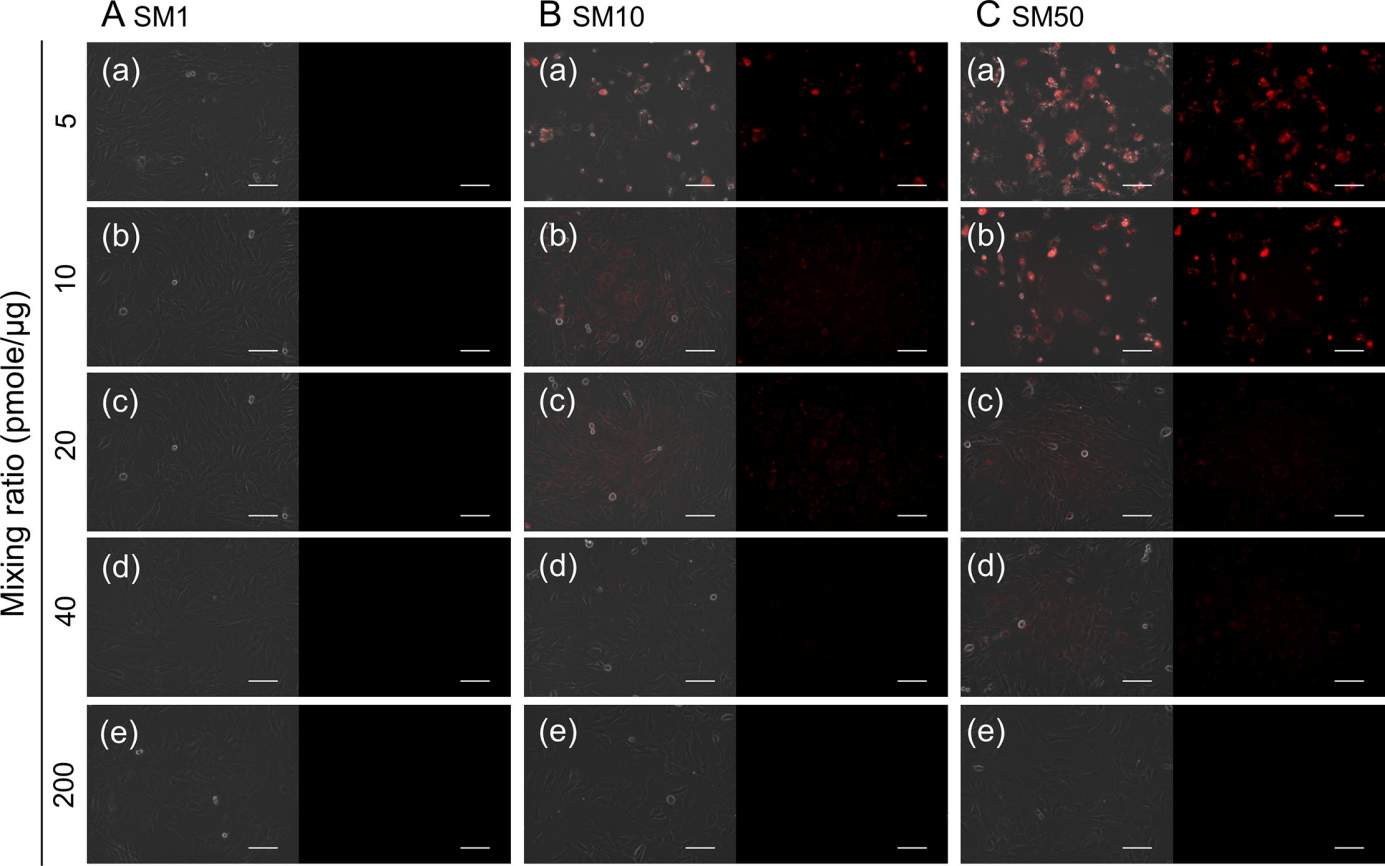
Fluorescent microscopic images of cells incubated with complexes prepared at various conditions. The cells were incubated with SM1 (A), SM10 (B), and SM50 complexes (C) prepared at mixing ratios of 5 (a), 10 (b), 20 (c), 40 (d), and 200 pmole MB/μg cationized gelatin (e). The GAPDH MB concentration is 200 nM. After incubation with complexes for 24 hr, the images were taken. Left panel shows the merged images of phase contrast microscopy and MB fluorescence, while the right panel shows the MB fluorescence. Scale bar is 100 μm.

Finally, the ability of complexes to detect the mRNA was evaluated. GAPDH mRNA was selected as the endogenous target because of the constant expression in each cell. Fig 4 shows the fluorescent microscopic images of cells incubated with SM1, SM10 and SM50 complexes prepared at various mixing ratios. The fluorescence of SM1 complexes was hardly detected at any mixing ratio (Fig 4A). For the complexes prepared with SM10 and SM50 at 5 pmole MB/μg cationized gelatin (Fig 4B (a) and 4C (a)) and with SM50 at 10 pmole MB/μg cationized gelatin (Fig 4C (b)), strong fluorescent spots were observed in dead cells. When compared at the same mixing ratio, the fluorescence tended to become strong with an increase of the cationization extent.

**Fig 5:**
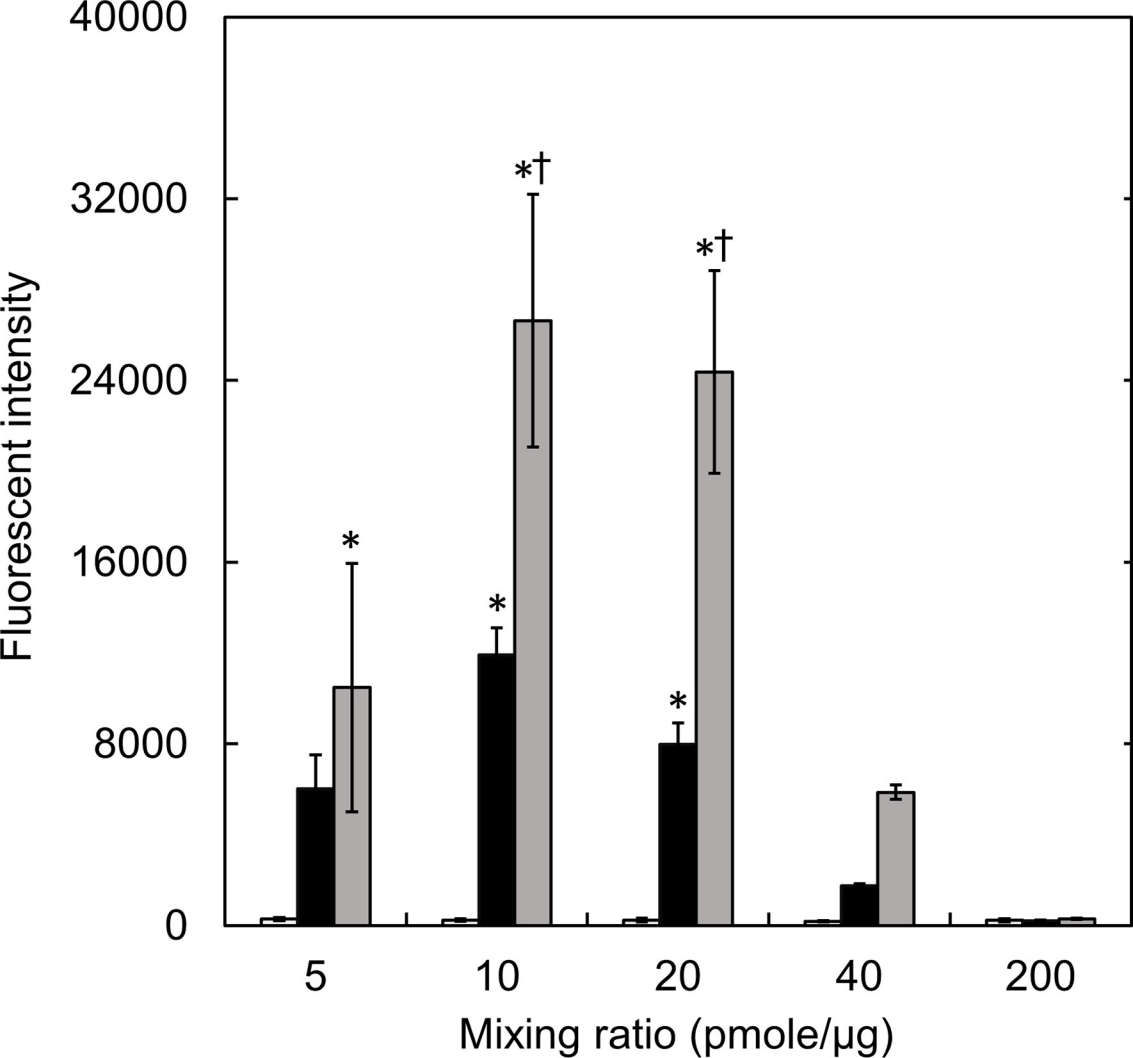
Flow cytometric analysis of cells incubated with complexes prepared at various conditions. The cells were incubated with SM1 (□), SM10 (■), and SM50 complexes (■) prepared at different mixing ratios. The GAPDH MB concentration is 200 nM. *, p < 0.05; significant against the fluorescent intensity of SM1 complexes at the corresponding mixing ratio. †, p < 0.05; significant against the fluorescent intensity of SM10 complexes at the corresponding mixing ratio.

Fig 5 shows the flow cytometric analysis of complexes after the elimination of dead cells. The fluorescent intensity became stronger as the cationization extent increased although this tendency was not seen at the mixing ratio of 200 pmole MB/μg cationized gelatin. Considering the cytotoxicity and fluorescent intensity, the complexes prepared at 20 pmole/μg was used in the following experiments.

**Fig 6:**
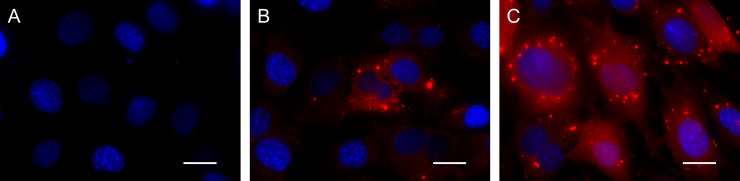
Fluorescent microscopic images of cells incubated with GAPDH MB complexes prepared at 20 pmole/μg. The cells were incubated with SM1 (A), SM10 (B), and SM50 (C) complexes prepared at the mixing ratio of 20 pmole MB/μg cationized gelatin. The GAPDH MB concentration is 200 nM. After the incubation with complexes for 24 hr, the images were taken. Red: GAPDH MB. Blue: nuclei. Scale bar is 20 μm.

Fig 6 shows the fluorescent microscopic images of cells incubated with the SM1, SM10 and SM50 complexes prepared at 20 pmole MB/μg cationized gelatin. The fluorescence of SM10 and SM50 complexes was observed in the cells, whereas that of SM1 complex was hardly observed. The fluorescent intensity and the number of fluorescent cells incubated with SM50 complex were larger than those incubated with the SM10 complex.

### Imaging of the EGFP mRNA exogenously transfected

**Fig 7:**
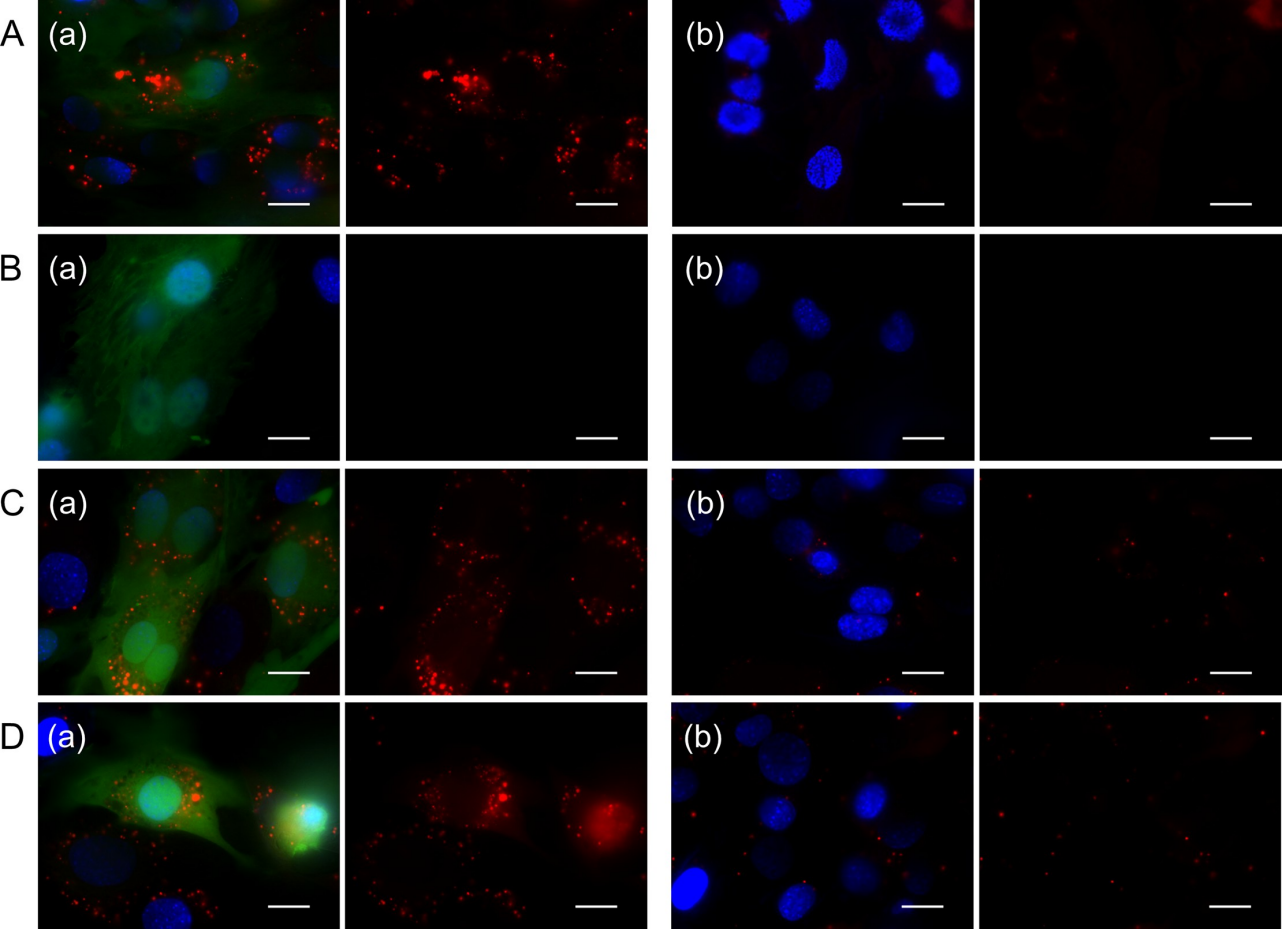
Fluorescent microscopic images of cells incubated with EGFP MB complexes prepared at 20 pmole/μg. The cells were incubated with HVJ-E (A), SM1 (B), SM10 (C), and SM50 (D) complexes prepared at the mixing ratio of 20 pmole MB/μg cationized gelatin. The EGFP MB concentration is 200 nM. After incubation with complexes, (a) EGFP mRNA was transfected by Lipofectamine^Ⓡ^ MessengerMAX^TM^ 3 hr later or (b) not transfected. The fluorescent images were taken after the transfection for 36 hr. Red: EGFP MB. Green: EGFP. Blue: nuclei. The left panel shows the merged fluorescence, while the right panel shows the MB fluorescence. Scale bar is 20μm.

To further evaluate the function of complexes, the mRNA of EGFP was exogenously transfected to detect the MB visualization. Fig 7 shows the fluorescent microscopic images of cells with or without transfection of target EGFP mRNA after incubation with the SM1, SM10, and SM50 complexes prepared at 20 pmole/μg or HVJ-E complexes. For the HVJ-E complex, a stronger fluorescence was detected by the transfection of EGFP mRNA. The fluorescence of SM1 complex was hardly detected, irrespective of the EGFP mRNA transfection. On the contrary, for cells incubated with SM10 and SM50 complexes, a stronger fluorescence was detected by the transfection of EGFP mRNA. Fluorescence was hardly observed even though the EGFP mRNA was not transfected (Fig 7A(b), 7B(b), and 7C(b)).

## Discussion

The present study demonstrates that the preparation conditions of cationized gelatin-MB complexes affect their physicochemical properties. Especially, the cationization extent of cationized gelatin was essential. The cytotoxicity, cell internalization, and MB imaging were evaluated by the culture of cells with various complexes. The complexes of highly cationized gelatin at a low mixing ratio showed a high cytotoxicity. On the other hand, for the complexes, the amount of MB internalized into cells was large. This may be because the cationic extent was high or the mixing ratio was low. The fluorescent intensity of complexes in the cells, in other words, the MB detection efficacy, was the highest for the SM50 complex mixed at 20 pmole/μg. This MB detection was observed both the endogenous GAPDH mRNA and EGFP mRNA exogenously transfected. It is possible that this result is due to a balance of cytotoxicity and the MB amount internalized.

The percentage of amino groups introduced increased as the increase of spermine amount to the carboxyl groups in preparation. However, the percent introduced did not increase even when the molar ratio was higher than 50. This can be explained by the steric hindrance. It is likely that when the number of spermine conjugated with gelatin increased, new spermine molecules cannot introduce due to the steric hindrance. SM3, SM5, and SM20 were used to preliminarily evaluate both the hybridization and cytotoxicity (S1 and S2 Figs). The SM3 or SM5 and SM20 showed an intermediate results between SM1 and SM10, and between SM10 and SM50, respectively. These findings suggest that their properties change monotonically as the spermine introduction rate increases. Based on this, to comprehensively evaluate the effect of different spermine introduction extents on the properties of complexes, three types of SM1, SM10, and SM50 cationized gelatin composites with maximum, minimum, and medium percentages of spermine introduced were selected in this study.

It is apparent in Table 2 that significant change in the complexes size was not observed, irrespective of the mixing ratio. It is conceivable that only some portions of cationized gelatin contribute to the complexation of MB. Consequently, the mixing ratio would not affect the apparent size of complexes. On the other hand, the zeta potential of complexes was influenced by the preparation conditions to change the values (Table 3). This can be explained in terms of increased cationic nature of complexes and decreased amount of anionic MB associated with the complexes. The apparent size and zeta potential of complexes prepared at 200 pmole/μg were not measured. It might be possible that the complexes prepared at the mixing ratio of 200 pmole/μg were aggregated to each other by the presence of excessive MB in the solution. In addition, the aggregated complexes might have a heterogeneous size distribution with different surface charges. It is highly conceivable that this heterogeneous aggregation would prevent the accurate measurements of apparent size and zeta potential.

The fluorescent intensity in the hybridization assay of complexes depended on the percent introduced of spermine or the target nucleotides (Fig 1). As one possible reason that the fluorescent intensity was decreased as the increase in the percent introduce, the hybridization would be inhibited by the electrostatic interaction of MB with the strongly cationized gelatin. However, it should be noted that the fluorescent intensity of MB incubated with the specific sequence was significantly higher than that with the non-specific sequence for any type of complexes used.

The cell viability decreased as the decreased mixing ratio or the increased percentage of amino groups introduced (Fig 2). Since the same amount of MB was incubated with cells, the concentration of cationized gelatin increased with the decreased mixing ratio. Consequently, the cytotoxicity would become higher. At the cationized gelatin concentrations of 40, 40, and 20 μg/ml for SM50, SM10, and SM50, respectively, a significant cytotoxicity was observed. The cationic extent of complexes generally increases with an increase in the percent introduced. This will cause the decreased cell viability. The zeta potential of complexes became large for complexes with a decreased mixing ratio (Table 3). It is well known that highly cationic materials show a high cytotoxicity [33]. The result of this study is consistent with this report. Since the cell membrane is of anionic charge, it is likely that cationic materials are easily internalized with the cell membrane, leading to the subsequent internalization [34, 35]. The same influence of cationized extent on the amount of MB internalized was observed (Fig 3). It may be that the amount of complexes internalized is well corresponded with that of MB internalized, since the MB is always associated with the complex.

There was no fluorescence detection for the SM1 complex or complexes prepared at the high mixing ratios (Fig 4). Bright spots were observed for the SM50 complexes mixed at 5 or 10 pmole/μg and the SM10 complex mixed at 5 pmole/μg at death cells (Fig 4). It is apparent in Fig 3 that only the small amount of MB was internalized into cells. Taken together, we can say with certainty that the cell membrane is damaged, and consequently complexes are easily internalized into cells, leading to a strong fluorescence in cells.

The fluorescent intensity of MB internalized in the flow cytometry assay comes from only the living cells (Fig 5), since the death cells were removed before the assay. The fluorescent intensity of the SM50 complex was higher than that of the SM1 and SM10 complexes. The large amount of MB was internalized into cells for the SM50 complex (Fig 3). The MB presence in cells would increase the probability of MB reacting with the target mRNA, resulting in an enhanced MB fluorescence. For the complexes at low mixing ratios, the cytotoxicity was high (Fig 2). This is due to the high concentration of cationized gelatin, leading to the low fluorescent intensity. In other words, since the dead cells internalizing a large amount of complexes were removed, only the living cells which had internalized a small amount of complexes, were evaluated with the flow cytometry. On the other hand, for the complexes at high mixing ratios, the fluorescent intensity was low (Fig 5). Considering the balance between the cytotoxicity and the fluorescent intensity, it is concluded that the SM50 complex prepared at a mixing ratio of 20 pmole/μg is an optimal system to detect the intracellular mRNA in the condition of this study.

In the panels of Fig 6 for the SM10 and SM50 complexes, the MB was spotted in the cell. It has been demonstrated that the intracellular mRNA was observed as dots in single molecule fluorescence *in situ* hybridization (smFISH) [36].

In the experiment to detect exogenous EGFP mRNA, HVJ-E was used as a positive control. HVJ-E is the particle prepared by inactivating the Hemagglutinating Virus of Japan and has an inherent ability of membrane fusion [37]. Since it is neither infectious nor proliferative, it has been used as a non-viral transfection tool for the genetic materials [37–39]. Because of the membrane fusion ability of HVJ-E, the cargo was directly transfected into cytosol. In the case of HVJ-E complex, MB is inserted into cytoplasm, and hybridize with the target mRNA to allow the fluorescence detection. On the other hand, in the case of cationized gelatin-MB complexes, the mechanism of MB detection is considered as shown below. The complexes are generally internalized into cells by an endocytosis pathway similarly to other transfection reagents of cationic lipids [40]. Since the spermine conjugated to the gelatin facilitates the endosomal escape of complexes by the pH buffering effect of secondary amino groups in spermine [41], it is likely that the complexes were released in the cytosol and consequently hybridize with the target mRNA. Incidentally, the weak fluorescence of MB was observed even in the cells that is not transfected EGFP mRNA (Fig 7). Moreover, in both the GAPDH and EGFP MB, fluorescent dots with different sizes and brightness and the diffused fluorescent background were observed. The reason why fluorescent dots varied and diffused background signal was observed is not clear at present. The background signal might be due to the non-specific fluorescence of MB (Fig 1). In the case of smFISH, the detection reactions of mRNA are performed in fixed and dead cells. On the other hand, MB were internalized into living cells in this study. It is highly possible that the local concentrations of intracellular target mRNA and MB are different between the living and fixed/dead cells in terms of spatio-temporal reaction. In addition, the degradation of MB by nucleases [42] and the non-specific interaction with intracellular proteins [43] will be possible reasons of these results. In the preliminary experiments (S3 Fig), the SM1, SM10, and SM50 complexes prepared at the mixing ratio of 20 pmole MB/μg cationized gelatin showed an increased stability against the nuclease (DNase I) with the increase of cationization extent. This can be due to the limited access of DNase I to the MB incorporated in the complexes as well as the inhibited recognition of MB as a substrate of the nuclease. In addition, it has been demonstrated that incorporation in the cationized gelatin nanospheres could prevent MB from the degradation by DNase I [30]. Therefore, in the case of complexes prepared in this study, the anti-nuclease stability would be enhanced compared with the free MB although further study is required to comprehensively investigate the incorporation ability of MB and the consequent stability for various complexes prepared with different cationized gelatins and mixing ratios. Some modified MB, including 2’-O-methylated RNA [44], locked nucleic acids (LNA) [44], and protein nucleic acids (PNA) [45], have been reported to be effective in avoiding the false-positive signal of MB. In addition to the carrier design of MB, chemical modifications of nucleic acids themselves would be utilized to further increase the specificity of MB and detection accuracy of target mRNA. In addition to the optimization of materials, it is also important for the accurate fluorescent imaging to consider the imaging methods to take the optical sections of cells for the reduction of background signals. Therefore, we tried the cross-section imaging of cells by use of fluorescent microscopy with an optical sectioning module. The optical sections of cells were taken based on the structured illumination in a grid pattern and the projection onto only the focused area of samples [46]. In this experiments, the dot-like fluorescence of MB with relatively low background signal was observed (S4 Fig) compared with the conventional fluorescent images (Fig 6). It is concluded that the combination of optical sectioning imaging and the preparation condition of materials optimized in this study would be potentially beneficial for the further improvement of mRNA imaging technology.

In addition to gelatin, various materials are known as carriers to incorporate nucleic acid substances into cells [47–52]. In these studies, the cytotoxicity or transfection efficiency is evaluated based on the N/P ratio; the molar ratios of the amino groups of carrier materials to the phosphate groups of nucleic acids, as a main factor. The cytotoxicity and transfection efficacy increase as the N/P ratios increase. This is because the complex is easily internalized into cells, since the electrostatic interaction between the complex and the cell membrane becomes stronger [48]. In addition, it is reported that when the N/P ratio increases, the transfection efficiency will reach a maximum, and then decrease [50–52]. It is claimed that the interaction between the carrier and nucleic acid substance is too strong, which may inhibit the release of nucleic acid in cells [50]. Table 4 shows the N/P ratios of complexes used in this study. The cytotoxicity and fluorescent intensity of complexes used in this study are evaluated by the N/P ratio. The cytotoxicity increased with the increasing N/P ratios. When compared between the complexes prepared with the same cationized gelatin, the fluorescent intensity increased with an increase of the N/P ratio, reached a maximum value, and then decreased. The decrease of MB fluorescence can be explained in terms of the strong electrostatic interaction between the cationized gelatin and MB at higher N/P ratios as well as the decreased MB amount internalized into cells (Fig 3). The fluorescence was hardly detected with the SM1 complexes, irrespective of the N/P ratios. The fluorescent intensity was influenced by the N/P ratios, and also the amounts of the spermine introduced into gelatin.

**Table 4:** N/P ^a^ ratio of complexes prepared.

	N/P ratio of complexes			
Mixing ratio (pmole/μg)^b^	SM1	SM10	SM50	
5	2.3	4.0	4.8	
10	1.2	2.0	2.4	
20	0.58	1.0	1.2	
40	0.29	0.51	0.60	
200	0.058	0.10	0.12	

^a^ The moler ratio of the amino groups of cationized gelatin to the phosphate groups of MB.

^b^ The amount of MB per 1 μg of cationized gelatin.

In the present study, we focused on the fluorescent detection property of the carrier material-MB complexes. The complexes were prepared at various conditions and optimized to visualize both the intracellular endogenous and exogenous mRNA. The physicochemical and hybridization properties, cytotoxicity, the amount of MB internalized into cells, and the resulting fluorescent detection efficacy of MB, were greatly influenced by the preparation conditions of complexes. We believe that the findings in this study are informative for wide research fields, and will contribute to the design of carrier materials used for the delivery of nucleic acids, such as pDNA, siRNA, as well as MB. To establish the imaging methods with MB, it is technically necessary to improve the sequence specificity of MB by adjusting the nucleotide sequence and to develop MB that emits stronger fluorescence, in addition to optimize the preparation conditions of complexes. By establishing this imaging methods, it will be possible to evaluate the time course of biological function or the state of living cells in real time. This imaging method is not only useful for basic researches, but also applications to the fields of drug discovery, cancer diagnosis, and regenerative medicine including cell therapy.

## Conclusions

The present study demonstrates that the preparation conditions of complexes affected their physicochemical properties. Especially, the cationic extent of complexes is the main factor to modify the properties. Upon incubating KUM6 cells with complexes, the percent survival of cells was low for the complexes prepared with a high cationic extent at a low mixing ratio. On the other hand, the amount of MB internalized depended on the type of complexes and the mixing ratio. The highest fluorescent intensity of MB in cells was observed for the SM50 complex prepared at a medium mixing ratio. This can be explained by the balance between the cytotoxicity and the amount of MB internalized. In addition, the EGFP mRNA exogenously transfected was also detected by this complex system. In conclusion, the complexation design of cationized gelatin and MB is essential to visualize the intracellular in the living cells.

## Supporting information

S1 Fig Hybridization specificity of free MB and MB incorporated in complexes. The fluorescent intensity of SM3 (A), SM5 (B), and SM20 complexes (C) mixed with different concentrations of specific (□) and non-specific target oligonucleotides (■). The concentration of GAPDH MB is 100 nM. *, p < 0.05; significant against the fluorescent intensity of non-specific target at the corresponding concentration.
(TIF)

S2 Fig Viability of cells incubated with complexes prepared at different mixing ratios. The cells were incubated with 1, 5, 10, 20, and 40 μg/ml SM3 (A), SM5 (B), and SM20 complexes (C). The GAPDH MB concentration is 200 nM. The viability of cells incubated without complex is expressed as 100%. *, p < 0.05; significant against the percent survival of cells incubated without complexes at the corresponding mixing ratio.
(TIF)

S3 Fig Anti-nuclease stability of complexes. Free MB or SM1, SM10, and SM50 complexes prepared at 20 pmole/μg were incubated with 20U/ml of DNase I for 15 min at 37°C and measured the fluorescent intensity to evaluate the degradation level of MB. The MB degraded level of free MB without DNase I is expressed as 1. *, p < 0.05; significant between the two groups.
(TIF)

S4 Fig Cross-section images of cells incubated with GAPDH MB complexes prepared at 20 pmole/μg. The cells were incubated with SM1 (A), SM10 (B), and SM50 complexes (C) prepared at the mixing ratio of 20 pmole MB/μg cationized gelatin. The GAPDH MB concentration is 200 nM. After the incubation with complexes for 24 hr, the cross-section images were taken. Red: GAPDH MB. Blue: nuclei. Scale bar is 20 μm.
(TIF)
