# Supplementary figures and images for: Complexation design of cationized gelatin and molecular beacon to visualize intracellular mRNA
Source: PLoS One. 2021 Jan 25;16(1):e0245899. doi: 10.1371/journal.pone.0245899 (PMC7833158; doi:10.1371/journal.pone.0245899)

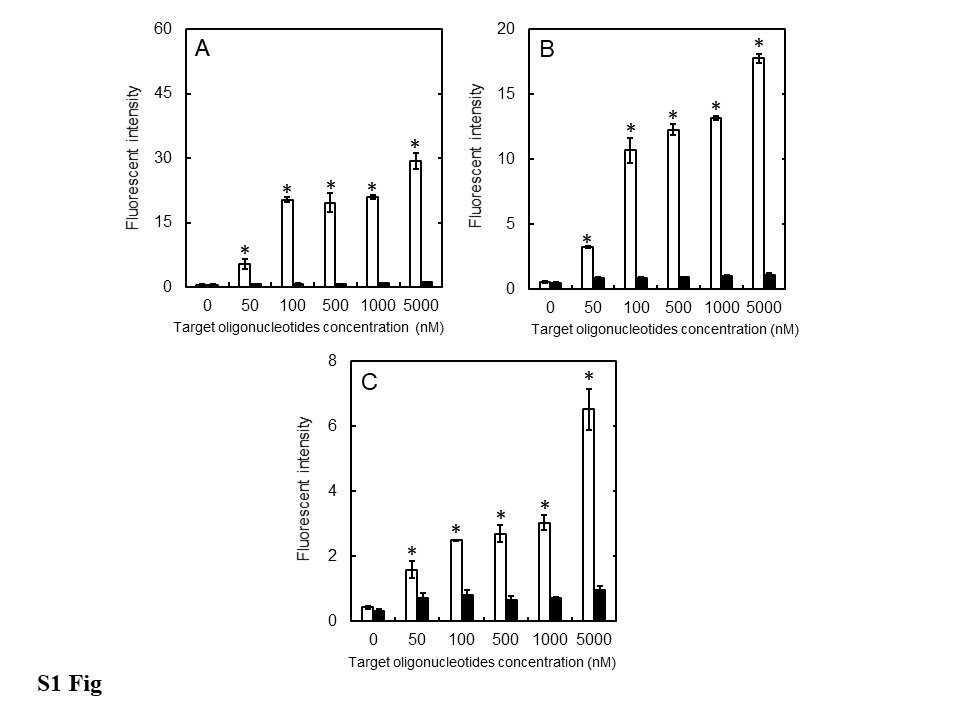

Supplement: S1 Fig — The fluorescent intensity of SM3 (A), SM5 (B), and SM20 complexes (C) mixed with different concentrations of specific (□) and non-specific target oligonucleotides (■). The concentration of GAPDH MB is 100 nM. *, p < 0.05; significant against the fluorescent intensity of non-specific target at the corresponding concentration. (TIF) [file pone.0245899.s001.tif]

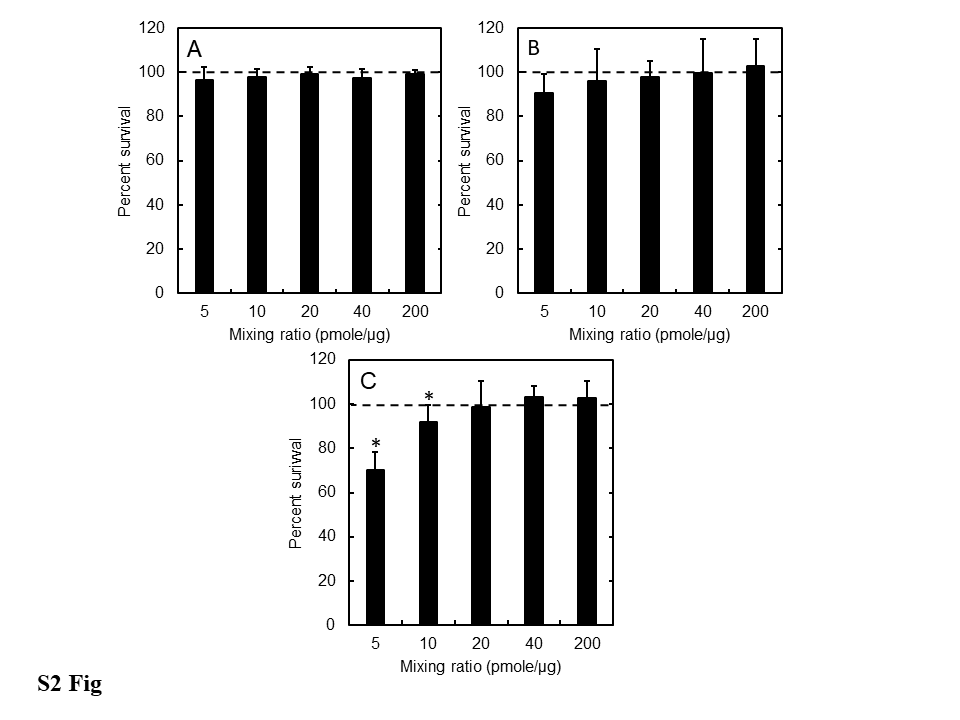

Supplement: S2 Fig — The cells were incubated with 1, 5, 10, 20, and 40 μg/ml SM3 (A), SM5 (B), and SM20 complexes (C). The GAPDH MB concentration is 200 nM. The viability of cells incubated without complex is expressed as 100%. *, p < 0.05; significant against the percent survival of cells incubated without complexes at the corresponding mixing ratio. (TIF) [file pone.0245899.s002.tif]

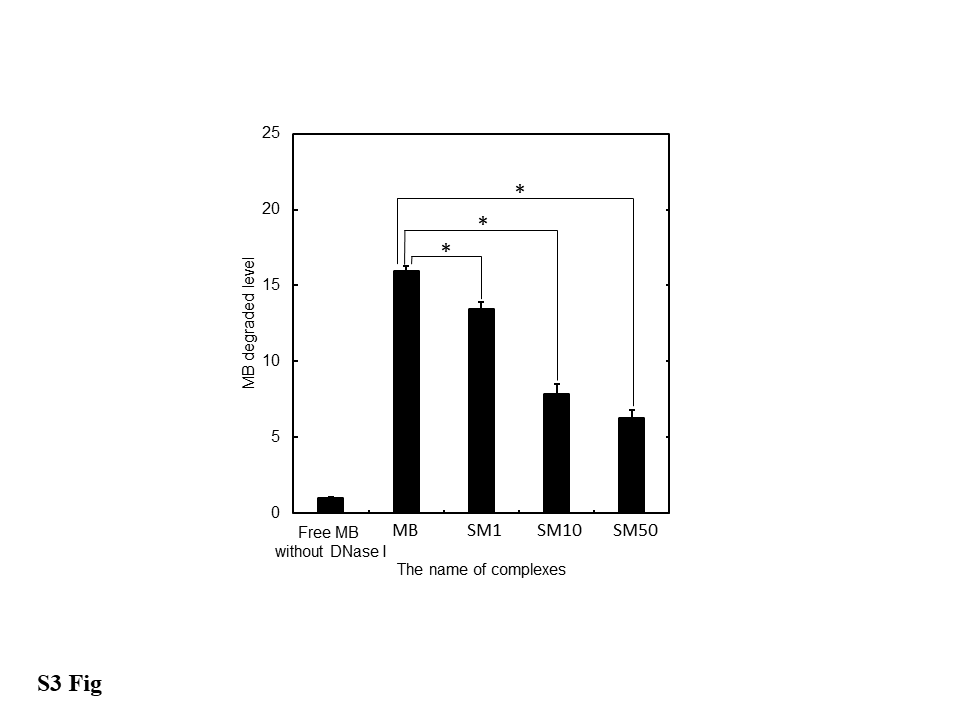

Supplement: S3 Fig — Free MB or SM1, SM10, and SM50 complexes prepared at 20 pmole/μg were incubated with 20U/ml of DNase I for 15 min at 37°C and measured the fluorescent intensity to evaluate the degradation level of MB. The MB degraded level of free MB without DNase I is expressed as 1. *, p < 0.05; significant between the two groups. (TIF) [file pone.0245899.s003.tif]

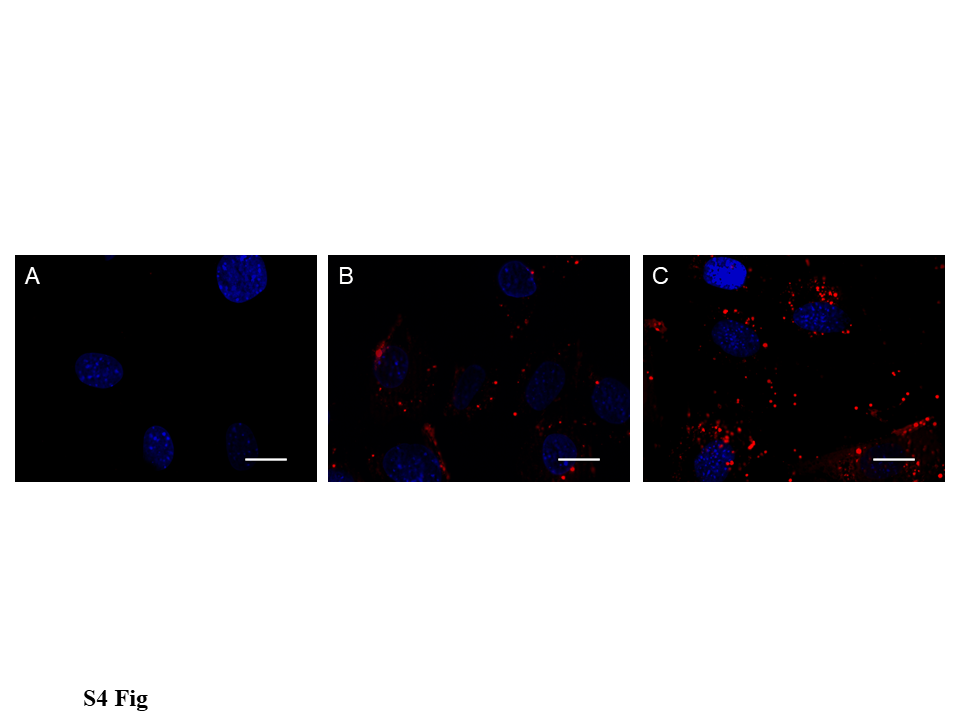

Supplement: S4 Fig — The cells were incubated with SM1 (A), SM10 (B), and SM50 complexes (C) prepared at the mixing ratio of 20 pmole MB/μg cationized gelatin. The GAPDH MB concentration is 200 nM. After the incubation with complexes for 24 hr, the cross-section images were taken. Red: GAPDH MB. Blue: nuclei. Scale bar is 20 μm. (TIF) [file pone.0245899.s004.tif]
